# Effects of the Modified DASH Diet on Adults With Elevated Blood Pressure or Hypertension: A Systematic Review and Meta-Analysis

**DOI:** 10.3389/fnut.2021.725020

**Published:** 2021-09-07

**Authors:** Ru Guo, Nian Li, Rong Yang, Xiao-Yang Liao, Yu Zhang, Ben-Fu Zhu, Qian Zhao, Lingmin Chen, Yong-Gang Zhang, Yi Lei

**Affiliations:** ^1^International Medical Center/Department of General Practice and National Clinical Research Center for Geriatrics, West China Hospital, Sichuan University, Chengdu, China; ^2^Department of Medical Administration, West China Hospital, Sichuan University, Chengdu, China; ^3^Department of Anesthesiology and National Clinical Research Center for Geriatrics, West China Hospital, Sichuan University and The Research Units of West China, Chinese Academy of Medical Sciences, Chengdu, China; ^4^Department of Periodical Press and National Clinical Research Center for Geriatrics, West China Hospital, Sichuan University, Chengdu, China; ^5^Chinese Evidence-Based Medicine Center, West China Hospital, Sichuan University, Chengdu, China

**Keywords:** dietary approaches to stop hypertension, hypertension, randomized controlled trial, meta-analysis, systematic review

## Abstract

**Background:** The modified Dietary Approaches to Stop Hypertension (DASH) diet was a potentially effective treatment for pre-hypertensive and hypertensive patients. The evidence for the effect of the modified DASH diet on blood pressure reduction was inconsistent. The study was designed to assess the effects of the modified DASH diet on blood pressure (BP) in hypertensive and pre-hypertensive adults.

**Methods:** We searched Medline, Embase, CENTRAL, CNKI, VIP, Wanfang Data, SINOMED, Google Scholar, the World Health Organization's International Clinical Trials Registry Platform, and Clinicaltrials.gov from inception to July 1st, 2021. Randomized controlled trials (RCTs) assessing the effects of the modified DASH diet on systolic and diastolic BP, cardiovascular risk factors (body weight, body mass index, waist circumference, fasting glucose, blood lipids), cardiovascular events, and all-cause mortality were included. Statistical analysis was performed using Stata software. Risk of bias was assessed with the Cochrane tool and quality of evidence with GRADE.

**Results:** A total of 10 RCTs were included. Compared with control diet, the modified DASH diet could reduce mean systolic (−3.26 mmHg; 95% confidence interval −5.58, −0.94 mmHg; *P* = 0.006) and diastolic (−2.07 mmHg; 95% confidence interval −3.68, −0.46 mmHg; *P* = 0.01) BP. Compared with the controlling diet, the modified DASH diet could reduce systolic BP to a greater extent in trials with a mean baseline BP ≥ 140/90 mmHg compared with <140/90 mmHg. Diastolic BP reduction was greater when the mean body mass index was ≥30 kg/m2 than <30 kg/m^2^. Diastolic BP reduction was more marked in trials with a follow-up time of >8 weeks compared with ≤8 weeks. The modified DASH diet could affect mean waist circumference (difference: 1.57 cm; 95% confidence interval −2.98, −0.15) and triglyceride concentration (difference: 1.04 mol/L; 95% confidence interval −1.47, −0.60).

**Conclusions:** The modified DASH diet can reduce BP, waist circumference, and triglyceride concentration in hypertension patients. A higher baseline BP is associated with more marked systolic and diastolic BP reduction.

**Systematic Review Registration:** PROSPERO registration number: CRD42020190860.

## Introduction

Hypertension, a primary risk factor for cardiovascular disease, is the leading cause of premature death worldwide and accounts for ~12.8% of global deaths (1–3). Systolic BP (SBP) or diastolic BP (DBP) increased by 20/10 mmHg will increase the risk of cardiovascular diseases by two times (4, 5). However, <20% of patients with hypertension achieve effective BP control (1). Therefore, lifestyle modifications, including a healthy diet, are recommended as potential therapeutic strategies (6–8).

The Dietary Approaches to Stop Hypertension (DASH) diet was the most typical dietary treatment strategy for BP control. It was abundant in fruits, vegetables and low-fat dairy products, with more fish, nuts and legumes, and a moderate sodium restriction (9). It reduced SBP and DBP by 5.5 mmHg and 3.0 mmHg in the first DASH clinical trial 20 years ago (10). DASH diet was effective in the reduction of BP and other cardiovascular risk factors, including blood glucose, blood lipids, body weight, and waist circumference (10–13). Hence, it was recommended in the treatment of hypertension (10–13). However, poor adherence was the main barrier, and only a small population fully adhered to the diet (3). Inappropriate translation, low spreading velocity, difficulty to follow, and contradiction with dietary culture were the main reasons (14–17).

The modified DASH diet, which expanded or reduced nutrient composition based on the original DASH diet, was one solution to poor adherence. It reduced the sodium level of DASH diet (6–3 g) to enhance the antihypertensive effect in DASH-Sodium research, which was the first study to modify the DASH diet (7). Many studies have been performed to assess the efficacy of modified DASH for hypertension patients (18–20). However, the results were inconsistent. Some studies reported that the modified DASH diet could reduce BP (18–20), body mass index (BMI) (19), blood sugar (18), and lipidemia (21); while other studies suggested the modified DASH diet might increase BP (22), blood sugar (18), and insulin resistance (23). Thus, we conducted a systematic review and meta-analysis to assess the efficacy of modified DASH diet for hypertension patients.

## Methods

### Study Registration and Reporting Guideline

The protocol was registered on PROSPERO (CRD4201007296). It was reported according to Preferred Reporting Items for Systematic Reviews and Meta-Analyses (PRISMA) guidelines (Supplemental Material 1) (24).

### Inclusion Criteria and Exclusion Criteria

The inclusion criteria were as follows: (1) study design: published and ongoing randomized controlled trials(RCTs); (2) participants: adults ≥18 years with mean SBP and DBP ≥130 or ≥80 mmHg; (3) intervention: advised or administered any type of modified DASH diet, either as a sole intervention or in combination with other interventions (e.g., exercise), and interventions described as “DASH-Sodium,” “DASH-Plus,” or “WELL diet” were included when they modified species or weight with foods in the DASH diet; (4) comparison: regular diet, traditional diet, typical American diet, or any other diet not related to DASH; (5) outcomes: primary outcomes included SBP and DBP, and secondary outcomes included cardiovascular risk factors [body weight, BMI, waist circumference, fasting glucose, total cholesterol (TC), triglycerides (TG), low-density lipoprotein (LDL), high-density lipoprotein (HDL)], cardiovascular events, and all-cause mortality. Searches were limited to the English and Chinese languages. Exclusion criteria were as follows: (1) study design: case reports, case series, observational studies, and animal research; (2) participants: individuals <18 years of age, pregnant women, and acute patients; (3) comparison: other different routes of modified DASH diet; (4) outcomes: outcomes with insufficient data, such as BP data at baseline or endpoint only.

### Databases and Search Strategy

We conducted a systematic search of several databases, including Medline (Ovid), EMbase (Ovid), Cochrane Central Register of Controlled Trials, CNKI, VIP, WanFang Data and SINOMED. The initially search was performed on October, 2020, and the updated search was performed on July 1st, 2021. We also searched Google Scholar, ClinicalTrials.gov, and the WHO's International Clinical Trials Registry Platform to identify ongoing trials. The following search terms were used: (DASH diet OR dietary approaches to stop hypertension) AND (hypertension OR blood pressure OR high blood pressure OR increased blood pressure OR elevated blood pressure) (Supplemental Material 2). Additionally, to identify relevant studies, we manually screened references in the included studies.

### Study Selection

Two researchers independently screened the titles and abstracts of records and performed a full-text review to evaluate all potentially eligible studies. Disagreements were resolved by discussion with a third reviewer. The following data were extracted from each study: (i) author, country, language, and year of publication; (ii) methods (study design, study analysis, sample sizes for both interventions and controls); (iii) risk of bias assessment; (iv) sample characteristics for both interventions and controls separately (gender ratio, mean age, ethnic background, mean baseline BP, mean BMI, specific measures and sodium intake of interventions and controls, methods of modified diet, follow-up duration, hypertension prevalence, anti-hypertension treatment, energy restriction of interventions). If the same dataset had been published more than once, we included the study with the largest number of participants or the best complete findings.

### Data Extraction

Two researchers independently extracted data from the included studies using a standard data extraction form. Disagreements were resolved by consensus or by a discussion with a third reviewer.

### Assessment of the Risk of Bias in Included Studies

Two researchers independently assessed the risk of bias of all included trials using the Cochrane risk bias tool (25). Quality of evidence were analyzed by using the grading of recommendations assessment, development, and evaluation (GRADE) approach.

### Statistical Analysis

The meta-analysis was conducted using Revman 5.3 software (26) and Stata software (version 12.0; StataCorp, College Station, TX). Mean differences (MDs) or standardized MDs (SMDs) before and after the intervention was obtained. Cochran's Q statistic (significance level, *P* < 0.10) and the *I*^2^ statistic were calculated to assess heterogeneity. We defined an *I*^2^ value of <50% as low heterogeneity and an *I*^2^ value >50% as high heterogeneity. A fixed-effects model was used when there was no significant heterogeneity; otherwise, the random-effects model was used. Sensitivity analysis was performed to analyze the stability of the results by sequentially omitting one study. When there were enough studies on BP outcomes, subgroup and meta-regression analyses were conducted to investigate the differential effects of the modified DASH diet on hypertension and to explore heterogeneity. These factors included age, gender, baseline BP (<140/90 or ≥140/90 mmHg), BMI (<30 or ≥30 kg/m^2^), duration of follow up (≤8 or >8 weeks), sodium intake (≤1,500 or >1,500 mg/d), control measures (healthy diet or common diet). Publication bias was assessed using visual inspection of funnel plots. A *P* < 0.05 was considered statistically significant.

## Results

### Eligible Studies and Study Characteristics

**Figure 1:**
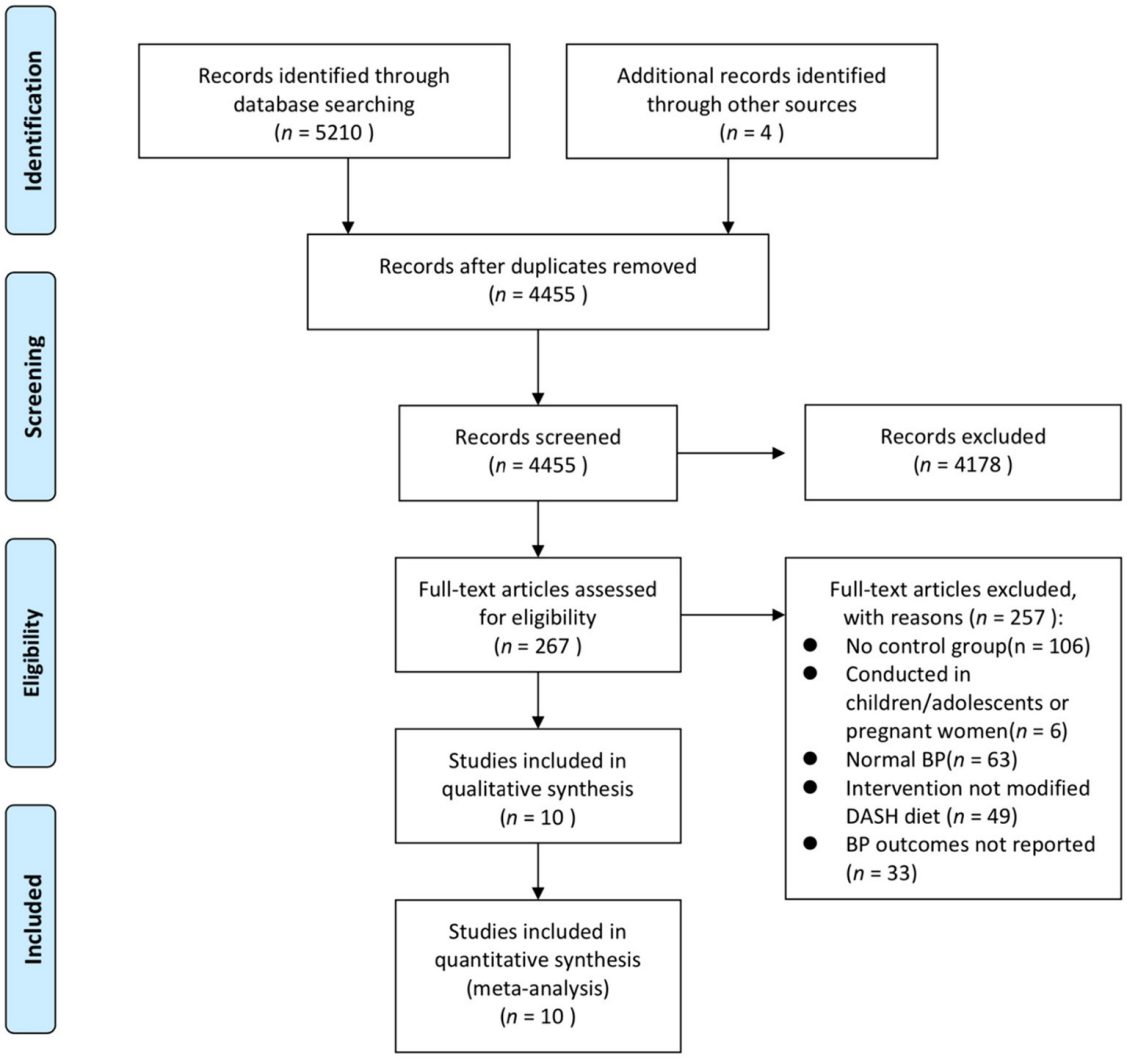
PRISMA flowchart of the included studies. BP, blood pressure; DASH, Dietary Approaches to Stop Hypertension; RCT, randomized controlled trial.

We initially identified 5,210 results through the literature search, with four studies identified through manual screening of reference lists from the included studies. After removing duplicates, 4,455 titles and abstracts were screened, and 267 articles remained for full-text review. Finally, a total of 10 studies were included (Figure 1) (7, 16, 18–20, 22, 27–30).

**Figure 2:**
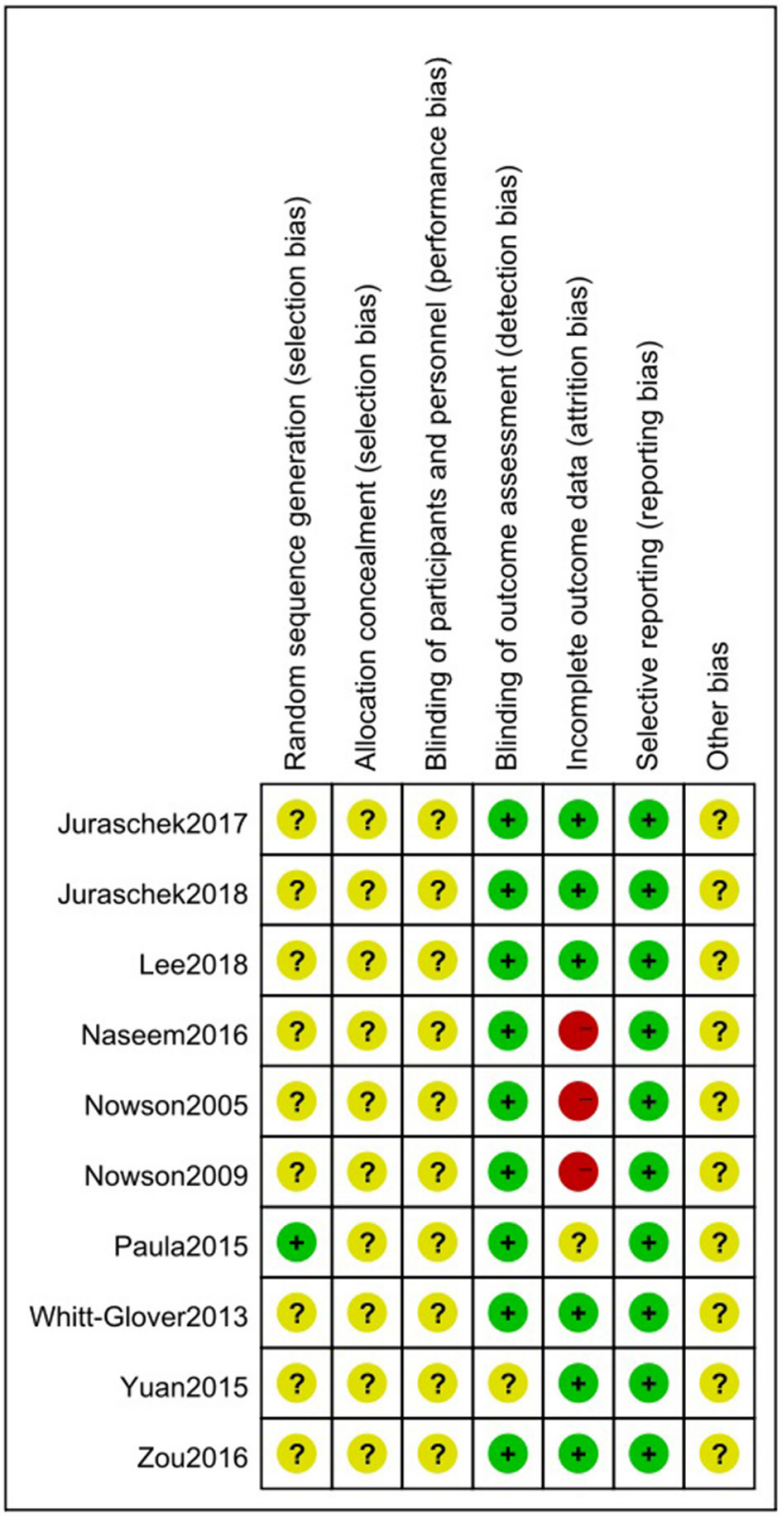
Risk of bias summary for eligible randomized controlled trials using the Cochrane risk-of-bias tool. The symbol “+” represents a low risk of bias, the symbol “–” represents a high risk of bias, and the symbol “?” represents an unclear risk of bias.

The studies included 2,416 participants with an age range from 45.10 to 62.20 years with a BP of 127/81 to 165/85 mmHg. Four studies were conducted in the USA and the others were from China, South Korea, Canada, Pakistan, and Brazil. Table 1 shows the characteristics of the included studies. More details are shown in Supplemental Materials 3, 4. The results of the risk bias assessment are shown in Figure 2 and Supplemental Material 5.

**Table 1:** Baseline and follow-up characteristics of included studies.

**Study**	**Country**	**Mean age (year)**	**Gender (Male, %)**	**Mean baseline BP (mmHg)**	**Mean BMI (kg/m^**2**^)**	**Intervention**	**Control**	**Subjects**		**FU (week)**	**Sodium intake (mg/d)**
								**I**	**C**		
Juraschek et al. (7)	American	48.3	43.2	134.80/85.70	29.20 ± 4.80	m-DASH	AD	192	186	4	3,000
Juraschek et al. (18)	American	59	29.8	131.50/77.20	34.70 ± 8.20	m-DASH	UD	60	57	8	NR
Lee et al. (22)	South Korea	45.1	78	134.90/86.40	25.20 ± 3.60	m-DASH	UD	30	28	8	NR
Naseem et al. (27)	Pakistan	53.4	51	128.30/84.30	27.60 ± 3.50	m-DASH	UD	710	782	5	1,500
Nowson et al. (28)	American	59.2	0	127.60/81.00	29.00 ± 3.00	m-DASH	HD	46	49	14	2,600–4,300
Nowson et al. (19)	Australia	47.9	100	129.40/80.60	30.40 ± 2.50	m-DASH	LFD	27	27	12	NR
Paula et al. (29)	Brazil	62.2	45	165.30/85.80	29.40 ± 3.10	m-DASH	DD	20	20	4	NR
Whitt-Glover et al. (16)	American	51.1	12	130.00/78.40	35.90 ± 7.10	m-DASH	CD	14	11	12	2,300
Zou et al. (20)	Canada	62	48	146.00/89.10	NR	m-DASH	UD	29	28	8	1,200–1,500
Yuan et al. (30)	China	NR	58	142.30/90.30	NR	m-DASH	CD	46	54	48	NR

*BMI, body mass index; BP, blood pressure; C, control; I, intervention; NR, not reported*.

### Effect on BP

**Figure 3:**
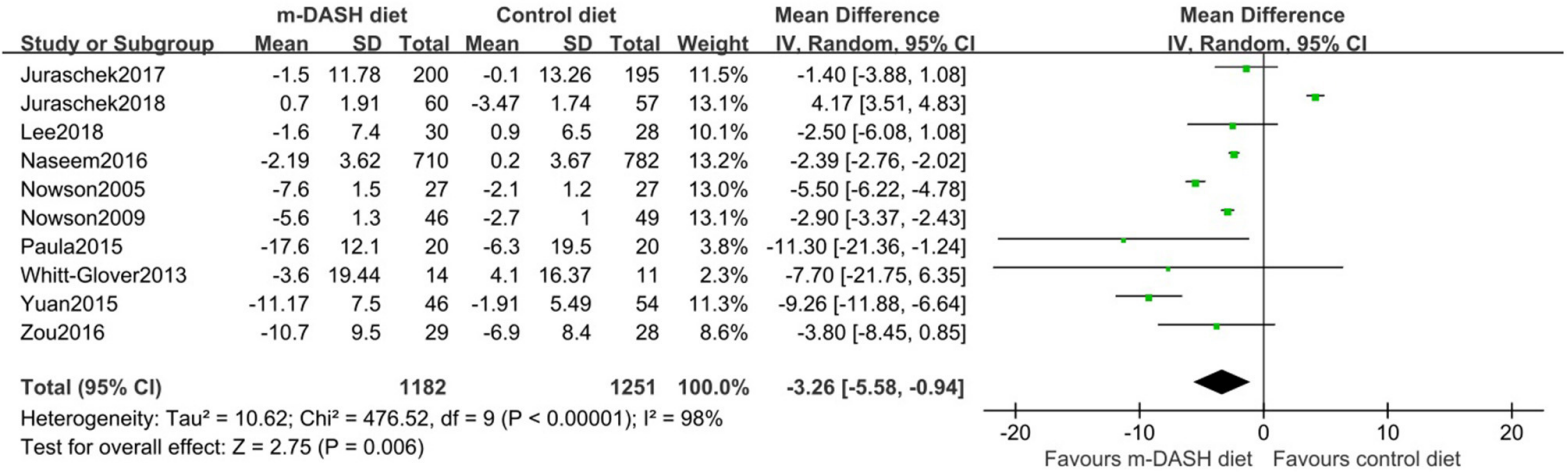
Forest plots of randomized controlled trials investigating the effects of the modified Dietary Approaches to Stop Hypertension (DASH) on systolic blood pressure (SBP).

**Figure 4:**
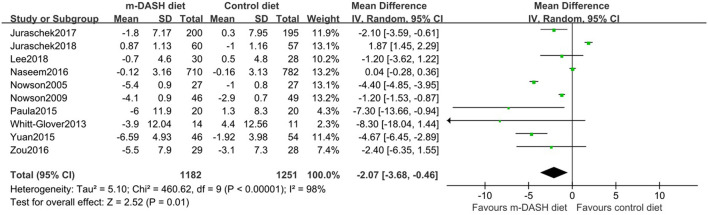
Forest plots of randomized controlled trials investigating the effects of the modified Dietary Approaches to Stop Hypertension (DASH) on diastolic blood pressure (DBP).

All ten trials reported BP before and after the intervention. Compared with the control diet, the modified DASH diet was associated with a significant reduction in SBP (MD: −3.26 mmHg; 95% CI −5.58, −0.94) and DBP (MD: −2.07 mmHg; 95% CI −3.68, −0.46) (Figures 3, 4). Subgroup analysis showed that the reduction in SBP and DBP was significantly greater in subgroups with baseline SBP/DBP values of ≥140/90 mmHg compared with <140/90 mmHg (SBP: *P* = 0.02; DBP: *P* = 0.01). Furthermore, DBP reduction was significantly greater in the subgroup with a longer follow-up period compared with a shorter follow-up period (*P* = 0.04). Additionally, DBP reduction was significantly greater in subgroup with a BMI ≥ 30 kg/m^2^ compared with <30 kg/m^2^ (*P* = 0.04). SBP and DBP reductions showed no statistic difference between subgroups aged 45–60 years and subgroups aged 60–74 years (SBP: *P* = 0.25; DBP: *P* = 0.26). No statistical difference was observed in subgroups with a sodium intake of >2,400 mg/d vs. ≤2,400 mg/d (SBP: *P* = 0.13; DBP: *P* = 0.26). No statistical difference was observed in subgroups with healthy diet vs. common diet (SBP: *P* = 0.40; DBP: *P* = 0.20). We found no significant interaction in prespecified subgroup meta-regressions for age, gender, baseline BP, BMI, duration of follow up, and sodium intake. However, control measures (healthy diet or common diet) appeared to be the main source of heterogeneity (SBP: *P* for interaction = 0.039; DBP: *P* for interaction = 0.048) (Supplemental Materials 6, 7).

### Effect on Cardiovascular Risk Factors

As to cardiovascular risk factors, the waist data from two RCTs showed that mean waist was 1.57 cm lower in people receiving modified DASH diet than in those receiving control diet (22, 29). Besides, two studies showed that the reduction in TG concentration was greater in the modified DASH diet group compared with the control group (MD: −1.04 mmol/L) (19, 29). However, another two studies showed no significant difference in body weight reduction between the modified DASH diet group and the control group (MD: 0.31 kg) (19, 22). BMI was also not significantly reduced (MD: 0.6 ± 2.55). Additionally, no reduction in TC (MD: −0.98 mmol/L), LDL (MD: −0.41 mmol/L), or HDL (MD: −1.13 mmol/L) concentrations were observed between the two groups (Table 2). Additionally, no reduction in TC or HDL concentrations were observed between the two groups (Table 2).

**Table 2:** Effect of the modified DASH diet in adults with increased BP for cardiovascular risk factors.

**Outcome**	**RCT (*n*)**	**Subjects (*n*)**	**SMD (95%CI)**	***I*** ^**2**^ **%**	***P***
Weight	2	112	−0.31[−0.61, −0.01]	0	0.05
Waist	2	98	−1.57 [−2.98, −0.15]	0	0.03
TC	3	1,586	−0.98 [−2.46, 0.50]	96	0.19
TG	2	94	−1.04 [−1.47, −0.60]	7	0.00001
LDL	3	1,586	−0.41 [−1.06, 0.24]	85	0.21
HDL	2	1,546	−1.13 [−3.41, 1.14]	98	0.33

*BP, blood pressure; DASH, Dietary Approaches to Stop Hypertension; RCT, randomized controlled trial; TC, total cholesterol; TG, triglyceride; LDL, low-density lipoprotein; HDL, high-density lipoprotein*.

### Cardiovascular Events and All-Cause Mortality

We could not conduct a meta-analysis or systematic review of cardiovascular events and all-cause mortality because of limited data.

### Quality of Evidence

Using the GRADE approach summary of evidence, the quality of evidence for the primary outcome was moderate (Supplemental Material 8).

### Publication Bias

**Figure 5:**
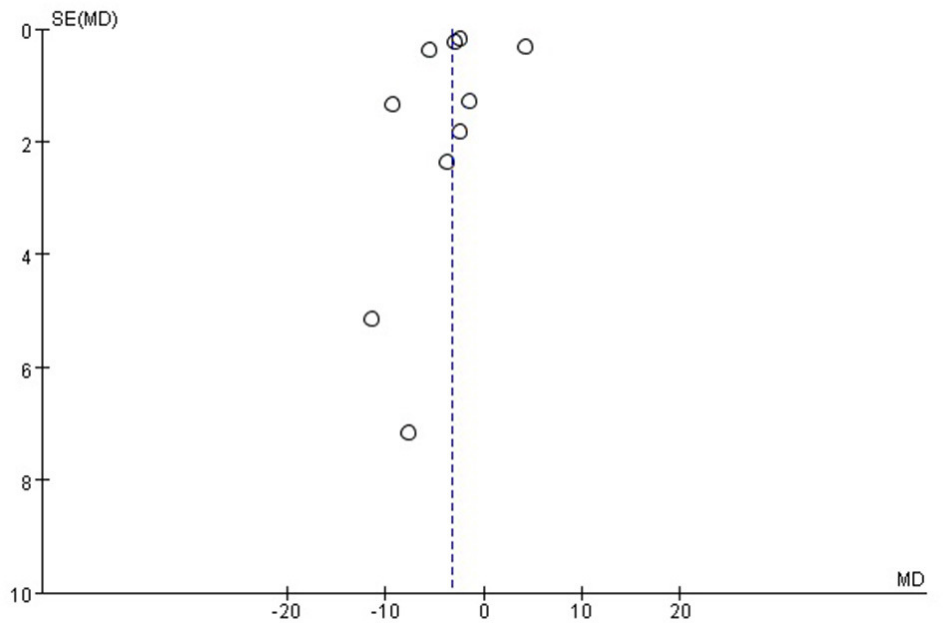
Funnel plot for the systolic blood pressure (SBP). Funnel plot with pseudo 95% confidence limits.

**Figure 6:**
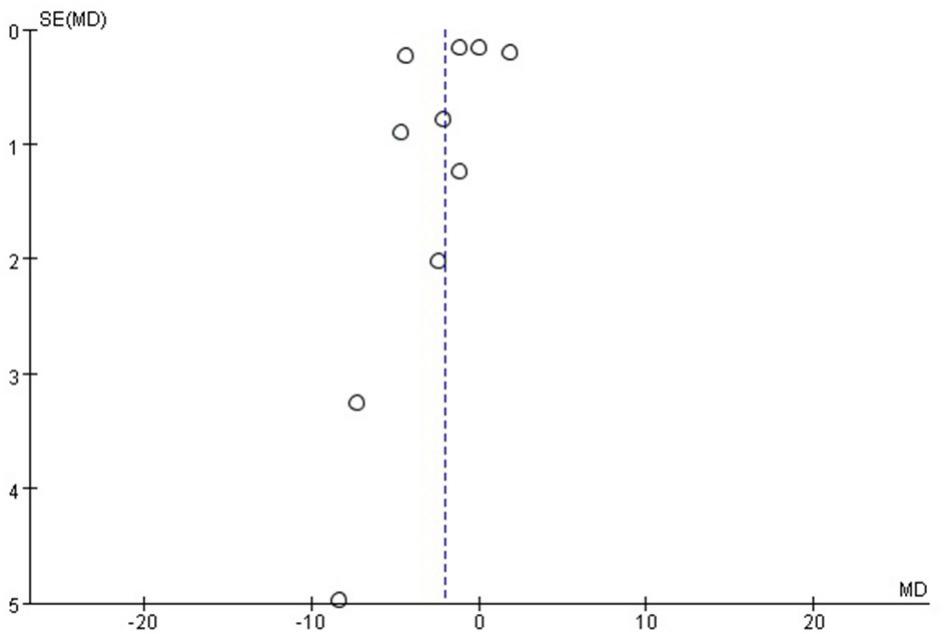
Funnel plot for the diastolic blood pressure (DBP). Funnel plot with pseudo 95% confidence limits.

As shown by the funnel plot analysis, no publication bias was found (Figures 5, 6).

### Sensitivity Analysis

Sensitivity analysis was performed to test the stability of the results by sequentially omitting one study. Our results showed that Yuan (2015) could influence the results of SBP (30).

## Discussion

In this study, we found that the modified DASH diet was associated with SBP and DBP reduction. Moreover, the modified DASH diet was associated with a reduction in waist circumference and TG concentration, but no statistical differences were found in weight or TC, LDL, and HDL concentrations. In subgroups of age, gender and control measures, analyses revealed that no association between the modified DASH diet and BP reduction. Although point estimates suggested that a higher daily sodium intake enhanced the BP-lowering effect, subgroup analyses did not show statistically significant differences. Furthermore, our finding indicated that DBP reduction was more prominent in groups with a more extended follow-up period or higher BMI. Moreover, SBP and DBP reduction were more pronounced in groups with a baseline SBP/DBP of ≥140/90 mmHg compared with < 140/90 mmHg. The modified DASH diet might have greater positive or negative effects in combination with antihypertensive treatment or have higher accordance or provision of different interventions, such as dietary advice or food supplements, however, we did not have sufficient data for these analysis.

Like previous studies (31–33), we found that the modified DASH diet was significantly associated with reductions in SBP, DBP, and cardiovascular risk factors. However, some discrepancies in the degree of BP and cardiovascular risk factor reduction were found. We found no association between the modified DASH diet and BP reduction in subgroups stratified by sodium intake and follow-up period.

The effect of the DASH diet on BP was investigated in previous meta-analyses (10, 34–36), which were consisted with our study. However, the DASH diet reduced SBP and DBP by 4.9–7.6 and 2.6–4.2 mmHg, respectively, which were more than our results (SBP: 3.26 mmHg; DBP: 2.07 mmHg). The reasons might be that we contained pre-hypertensive patients. In addition, subgroup analyses found that the mean BP reduction (SBP: 7.59 mmHg; DBP: 4.47 mmHg) in patients with a baseline SBP/DBP threshold of ≥140/90 mmHg was similar to the BP range in the previous studies. Furthermore, Filippou et al. (11) found that in adults with and without hypertension, the DASH diet could reduce SBP and DBP by 3.2 and 2.5 mmHg, respectively, which was similar to our study.

Wong et al. conducted a RCT at primary care institutions and showed that as follow-up time was extended, patients' dietary compliance and BP reduction decreased (37), which contradicted with our study findings. Most studies included in our study had a follow-up period of 4–12 weeks, which might have been too short to observe a decrease in dietary compliance. Thus, more studies with a longer follow-up time were still needed.

A study showed that more than 30% of hypertension cases were caused by excessive salt intake (38); thus, BP decreased with sodium intake decreased (33). However, contradictory to other studies (31), we found a lower sodium supplication could not lead to better BP reduction. This might because the modified DASH diet was strict sodium restriction, which was generally as low as 3,000 mg. That also suggested that too low sodium intake did not reduce BP further but might reduce adherence to dietary treatment.

The resutls of meta-regression showed that control measures (healthy diet or common diet) appeared to be the main source of heterogeneity for SBP and DBP. However, in our subgroup analysis, no matter compared with healthy diet or common diet, the modified DASH diet was associated with a significant reduction in SBP and DBP, and no statistical difference was observed in the two subgroups. Thus, even in the presence of heterogeneity, we could still conclude that the modified DASH diet could reduce BP.

A previous meta-analysis showed that the DASH diet was associated with an increased HDL concentration (17), and a reduction in waist circumference (32), BMI (31, 32), and TG, TC, and LDL concentrations (17). That was different from our findings, which did not show a reduction in waist circumference or TG concentration, and might be a consequence of the small sample size.

It was suggested that DASH diet could reduce BP and alleviate cardiovascular disease (2, 17, 39–42). Our study showed that the modified DASH diet had similar effects on BP reduction when compared with the DASH diet. Meanwhile, the modified DASH diet improved dietary adherence by enhancing the feasibility and acceptability of the diet. Hence, the modified DASH diet was an effective treatment for hypertensive patients.

There were some limitations to the study. First, some studies did not provide sufficient information about study design or other details. Second, the overall heterogeneity among studies was high and was partly downgraded by explanatory analyses. Third, although subgroup analyses were conducted across mean clinical thresholds in each separate trial, it was undetermined individual trial patients were above or below these preselected thresholds. Finally, the included study did not report or described the blinding process and methods in detail, thus no further analysis was performed.

## Conclusion

In conclusion, the current systematic review and meta-analysis demonstrates that a modified DASH diet can reduce BP in pre-hypertensive and hypertensive patients. A higher baseline BP is associated with more prominent systolic and diastolic BP reduction. Further large-sample and high-quality studies are needed to verify the above conclusions.

## Data Availability Statement

The original contributions presented in the study are included in the article/Supplementary Files, further inquiries can be directed to the corresponding authors.

## Author Contributions

YL and Y-GZ designed the study and edited the manuscript. RG, RY, X-YL, YZ, B-FZ, QZ, and LC searched the data, analyzed the data, drafted the manuscript. NL revised the manuscript. All authors approved the final version of the manuscript.

## Funding

This study was supported by the grant of the National Key Research and Development Program of China (No. 2017YFC0907303); Science & Technology Department of Sichuan province (No. 2020YFSY0014 and 2020YFS0186); National Clinical Research Center for Geriatrics, West China Hospital, Sichuan University (Z20191009).

## Conflict of Interest

The authors declare that the research was conducted in the absence of any commercial or financial relationships that could be construed as a potential conflict of interest.

## Publisher's Note

All claims expressed in this article are solely those of the authors and do not necessarily represent those of their affiliated organizations, or those of the publisher, the editors and the reviewers. Any product that may be evaluated in this article, or claim that may be made by its manufacturer, is not guaranteed or endorsed by the publisher.

## Abbreviations

DASH: The Dietary Approaches to Stop Hypertension
BP: blood pressure
RCT: randomized controlled trial
SBP: Systolic blood pressure
DBP: diastolic blood pressure
ISH: International Society of Hypertension
BMI: body mass index
PRISMA: Preferred Reporting Items for Systematic Reviews and Meta-Analyses
MDs: mean differences
SMDs: standardized mean differences
TG: Triglycerides
TC: Total cholesterol
LDL: Low-density lipoprotein
HDL: High-density lipoprotein.
