# Corrigendum: Effects of the Modified DASH Diet on Adults With Elevated Blood Pressure or Hypertension: A Systematic Review and Meta-Analysis

**DOI:** 10.3389/fnut.2021.778414

**Published:** 2021-10-05

**Authors:** Ru Guo, Nian Li, Rong Yang, Xiao-Yang Liao, Yu Zhang, Ben-Fu Zhu, Qian Zhao, Lingmin Chen, Yong-Gang Zhang, Yi Lei

**Affiliations:** ^1^International Medical Center/Department of General Practice and National Clinical Research Center for Geriatrics, West China Hospital, Sichuan University, Chengdu, China; ^2^Department of Medical Administration, West China Hospital, Sichuan University, Chengdu, China; ^3^Department of Anesthesiology and National Clinical Research Center for Geriatrics, West China Hospital, Sichuan University and The Research Units of West China, Chinese Academy of Medical Sciences, Chengdu, China; ^4^Department of Periodical Press and National Clinical Research Center for Geriatrics, West China Hospital, Sichuan University, Chengdu, China; ^5^Chinese Evidence-Based Medicine Center, West China Hospital, Sichuan University, Chengdu, China

**Keywords:** dietary approaches to stop hypertension, hypertension, randomized controlled trial, meta-analysis, systematic review

In the original article, there was a mistake in Figure 1 as published. The PRISMA flowchart in Figure 1 did not show the number of studies after the updated search. The corrected Figure 1 appears below.

**Figure 1:**
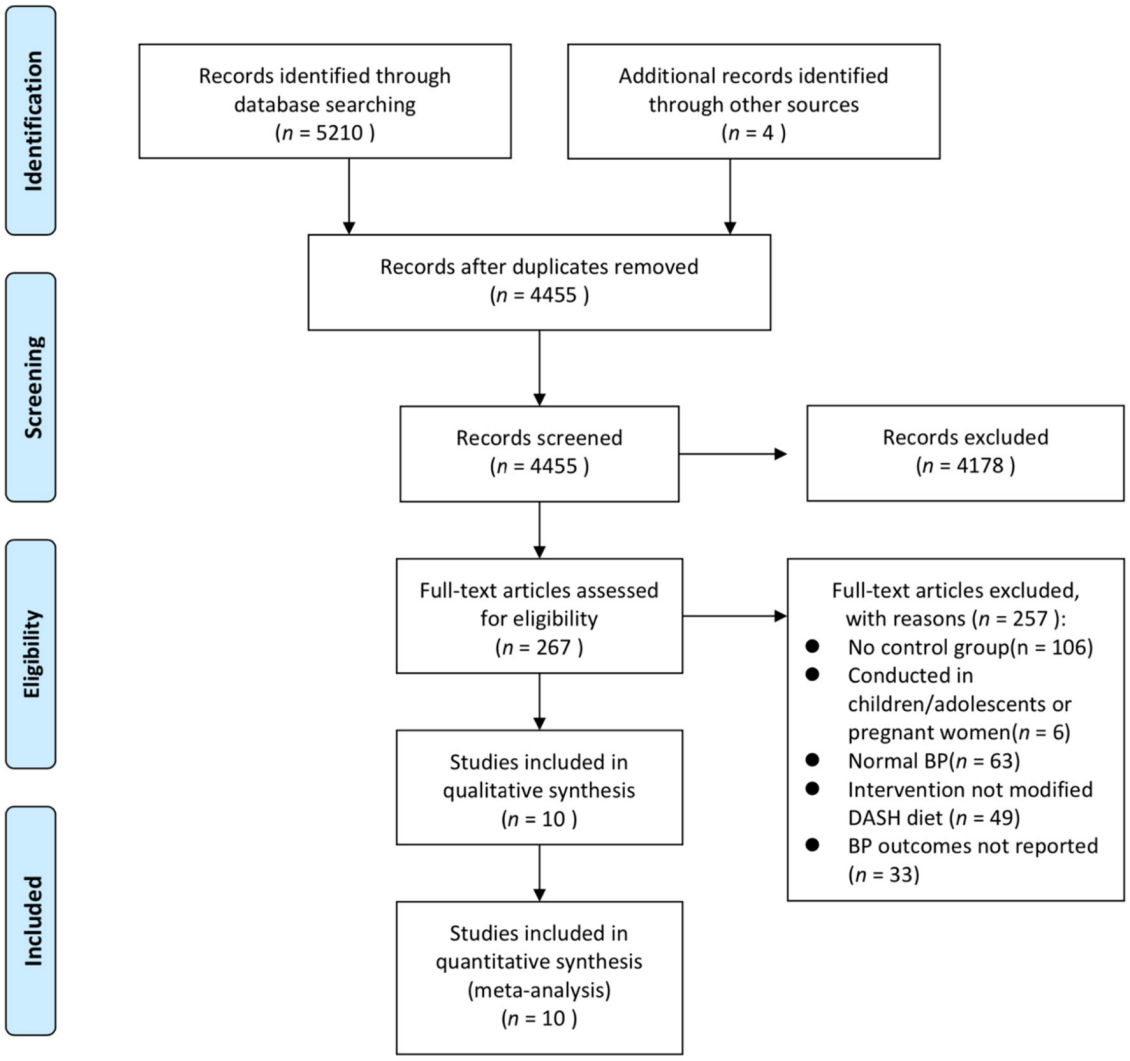
PRISMA flowchart of the included studies. BP, blood pressure; DASH, Dietary Approaches to Stop Hypertension; RCT, randomized controlled trial.

The authors apologize for this error and state that this does not change the scientific conclusions of the article in any way. The original article has been updated.

## Publisher's Note

All claims expressed in this article are solely those of the authors and do not necessarily represent those of their affiliated organizations, or those of the publisher, the editors and the reviewers. Any product that may be evaluated in this article, or claim that may be made by its manufacturer, is not guaranteed or endorsed by the publisher.

