# Supplementary material for: Effects of the Modified DASH Diet on Adults With Elevated Blood Pressure or Hypertension: A Systematic Review and Meta-Analysis
Source: Front Nutr. 2021 Sep 7;8:725020. doi: 10.3389/fnut.2021.725020 (PMC8452928; doi:10.3389/fnut.2021.725020)

Supplemental material 1: PRISMA checklist

| **Section/topic** | **#** | **Checklist item** | **Reported on page #** |
| --- | --- | --- | --- |
| **TITLE** | | |  |
| Title | 1 | Identify the report as a systematic review, meta-analysis, or both. | page. 1 |
| **ABSTRACT** | | |  |
| Structured summary | 2 | Provide a structured summary including, as applicable: background; objectives; data sources; study eligibility criteria, participants, and interventions; study appraisal and synthesis methods; results; limitations; conclusions and implications of key findings; systematic review registration number. | page. 1-2 |
| **INTRODUCTION** | | |  |
| Rationale | 3 | Describe the rationale for the review in the context of what is already known. | page. 2 |
| Objectives | 4 | Provide an explicit statement of questions being addressed with reference to participants, interventions, comparisons, outcomes, and study design (PICOS). | page. 2 |
| **METHODS** | | |  |
| Protocol and registration | 5 | Indicate if a review protocol exists, if and where it can be accessed (e.g., Web address), and, if available, provide registration information including registration number. | page. 2 |
| Eligibility criteria | 6 | Specify study characteristics (e.g., PICOS, length of follow-up) and report characteristics (e.g., years considered, language, publication status) used as criteria for eligibility, giving rationale. | page. 2 |
| Information sources | 7 | Describe all information sources (e.g., databases with dates of coverage, contact with study authors to identify additional studies) in the search and date last searched. | page. 2 |
| Search | 8 | Present full electronic search strategy for at least one database, including any limits used, such that it could be repeated. | page. 2  Supplemental material 2 |
| Study selection | 9 | State the process for selecting studies (i.e., screening, eligibility, included in systematic review, and, if applicable, included in the meta-analysis). | page. 3 |
| Data collection process | 10 | Describe method of data extraction from reports (e.g., piloted forms, independently, in duplicate) and any processes for obtaining and confirming data from investigators. | page. 3 |
| Data items | 11 | List and define all variables for which data were sought (e.g., PICOS, funding sources) and any assumptions and simplifications made. | page. 2-3 |
| Risk of bias in individual studies | 12 | Describe methods used for assessing risk of bias of individual studies (including specification of whether this was done at the study or outcome level), and how this information is to be used in any data synthesis. | page. 3 |
| Summary measures | 13 | State the principal summary measures (e.g., risk ratio, difference in means). | page. 3 |
| Synthesis of results | 14 | Describe the methods of handling data and combining results of studies, if done, including measures of consistency (e.g., I^2^) for each meta-analysis. | page. 3 |

| **Section/topic** | **#** | **Checklist item** | **Reported on page #** |
| --- | --- | --- | --- |
| Risk of bias across studies | 15 | Specify any assessment of risk of bias that may affect the cumulative evidence (e.g., publication bias, selective reporting within studies). | page. 3 |
| Additional analyses | 16 | Describe methods of additional analyses (e.g., sensitivity or subgroup analyses, meta-regression), if done, indicating which were pre-specified. | page. 3 |
| **RESULTS** | | |  |
| Study selection | 17 | Give numbers of studies screened, assessed for eligibility, and included in the review, with reasons for exclusions at each stage, ideally with a flow diagram. | page. 3  Figure 1 |
| Study characteristics | 18 | For each study, present characteristics for which data were extracted (e.g., study size, PICOS, follow-up period) and provide the citations. | page. 3  Table 1, Supplemental material 3 and 4 |
| Risk of bias within studies | 19 | Present data on risk of bias of each study and, if available, any outcome level assessment (see item 12). | page. 5  Figure 2, Supplemental material 5 |
| Results of individual studies | 20 | For all outcomes considered (benefits or harms), present, for each study: (a) simple summary data for each intervention group (b) effect estimates and confidence intervals, ideally with a forest plot. | page. 3  Figures 3, Figures 4 |
| Synthesis of results | 21 | Present results of each meta-analysis done, including confidence intervals and measures of consistency. | page. 3-4  Table 2 |
| Risk of bias across studies | 22 | Present results of any assessment of risk of bias across studies (see Item 15). | page. 5 |
| Additional analysis | 23 | Give results of additional analyses, if done (e.g., sensitivity or subgroup analyses, meta-regression [see Item 16]). | page. 3-4  Figures 5, Figures 6, Supplemental material 6, Supplemental material 7 |
| **DISCUSSION** | | |  |
| Summary of evidence | 24 | Summarize the main findings including the strength of evidence for each main outcome; consider their relevance to key groups (e.g., healthcare providers, users, and policy makers). | page. 5-7 |
| Limitations | 25 | Discuss limitations at study and outcome level (e.g., risk of bias), and at review-level (e.g., incomplete retrieval of identified research, reporting bias). | page. 7 |
| Conclusions | 26 | Provide a general interpretation of the results in the context of other evidence, and implications for future research. | page. 7 |
| **FUNDING** | | |  |
| Funding | 27 | Describe sources of funding for the systematic review and other support (e.g., supply of data); role of funders for the systematic review. | page. 7 |

Supplemental material 2: Search strategy of the study

Database: Embase（Ovid） <1990 to Present> July, 2021

1. DASH diet/

2. (DASH adj3 diet*).tw.

3. (DASH adj6 food*).tw.

4. (DASH adj6 nutrition*).tw.

5. (DASH adj6 pattern*).tw.

6. ( ("diet* pattern*" or "food* pattern*" or " eat* pattern*" ) adj2 (hypertension or "blood pressure")).tw.

7. dietary approaches to stop hypertension/

8. or/1-7

9. modified or modifying

10.8 and 9

11.exp hypertension/

12. hypertensi*.tw.

13. ((high or increased or elevated) adj2 blood pressure).tw.

14. Blood Pressure/

15. blood pressure.tw.

16. or/11-15

17. 10 and 16

18. (animal/ or nonhuman/) not human/

19. 17 not 18

Supplemental material 3: The characteristics of included studies.

| Study | Design | Blinded | Analysis | HTN prevalence,% | Anti-HTN treatment | CF | ER |
| --- | --- | --- | --- | --- | --- | --- | --- |
|  |  |  |  |  |  |  |  |
| Juraschek2017^[7]^ | P | Yes | PP | 41 | NR | Yes | Yes |
| Juraschek2018^[18]^ | P | NR | PP | NR | Yes | No | No |
| Lee2018^[22]^ | P | NR | ITT | NR | Yes | No | No |
| Naseem2016^[27]^ | P | NR | PP | 100 | Yes | No | Yes |
| Nowson2009^[28]^ | P | NR | PP | NR | No | No | No |
| Nowson2005^[19]^ | P | NR | PP | NR | Yes | No | No |
| Paula2015^[29]^ | P | NR | PP | 100 | Yes | No | Yes |
| WhittGlover2013^[16]^ | P | NR | ITT | 75 | Yes | No | No |
| Zou2016^[20]^ | P | Yes | PP | 100 | Yes | No | Yes |
| Yuan2015^[30]^ | P | NR | PP | 100 | Yes | No | No |

CF, controlled feeding; ER, energy restriction; HTN, hypertension; ITT, intention to treat; NR, not reported; P, parallel; PP, per protocol.

Supplemental material 4: The characteristics the DASH diet of included studies.

| Study | Methods of modified diet, and differences between modified DASH diet and classic DASH diet* |
| --- | --- |
| Juraschek2017^[7]^ | Based on the classic DASH diet, the DASH sodium diet has 3 sodium levels: low, medium, and high, which at 2100 kcal provided a target of 50, 100, and 150 mmol of sodium per day, and lower, similar, or higher than the classic DASH diet, respectively. |
| Juraschek2018^[18]^ | DASH-Plus diet was a tailored diet for home environment. It is based on the classic DASH diet and provides an allowance to purchases high potassium foods (fruits, vegetables, nuts, or dried beans) additionally. |
| Lee2018^[22]^ | Except for adherence to the principle of the classic DASH diet, the Korean modified DASH diet also advising the participants to abstain from adding table salt to their meals and adding salt during cooking, and abstain from consuming pickles and condiments. |
| Naseem2016^[27]^ | The diet plan was based on DASH diet, prepared to keep in view the eating habits of Pakistani population, and restrictive sodium intake to 1.5g/d (The standard DASH diet restrictive sodium intake to 2.3g/d). |
| Nowson2009^[28]^ | The vitality diet (VD), a low-sodium and dietary acid load DASH-type diet, which further reduce sodium and red meat consumption intake of the classic DASH diet. |
| Nowson2005^[19]^ | The DASH-type weight-loss diet (WELL diet) was modified from the US DASH diet, and further increasing fruit and vegetable intake and reducing fat intake, particularly saturated fat. |
| Paula2015^[29]^ | The diet based no the classic DASH diet eating plan, and was adapted to local dietary habits. |
| WhittGlover2013^[16]^ | The modified DASH diet was designed for community participants, to increase skills for preparing meals following DASH. It increases fruits and vegetables consumption to 9 to 12 servings per day (The standard DASH diet increase fruits and vegetables intake to 8 to 10 servings per day). |
| Zou2016^[20]^ | The DASHNa-CC diet was a culturally sensitive diet, which integrates traditional Chinese medicine food therapy into the current DASH and further reduces sodium. |
| Yuan2015^[30]^ | Chinese modified DASH diet was based on the classic DASH diet, further reducing sodium, increasing potassium, increasing calcium. |

*All modified DASH diet contains the principle of classic DASH diet: encouraged the consumption of fruits, vegetables, low-fat dairy foods, whole grains, lean meat, nuts, seeds, and beans, discouraged the intake of salt, fats, and sweets.

Supplemental material 5: Risk of bias assessment results


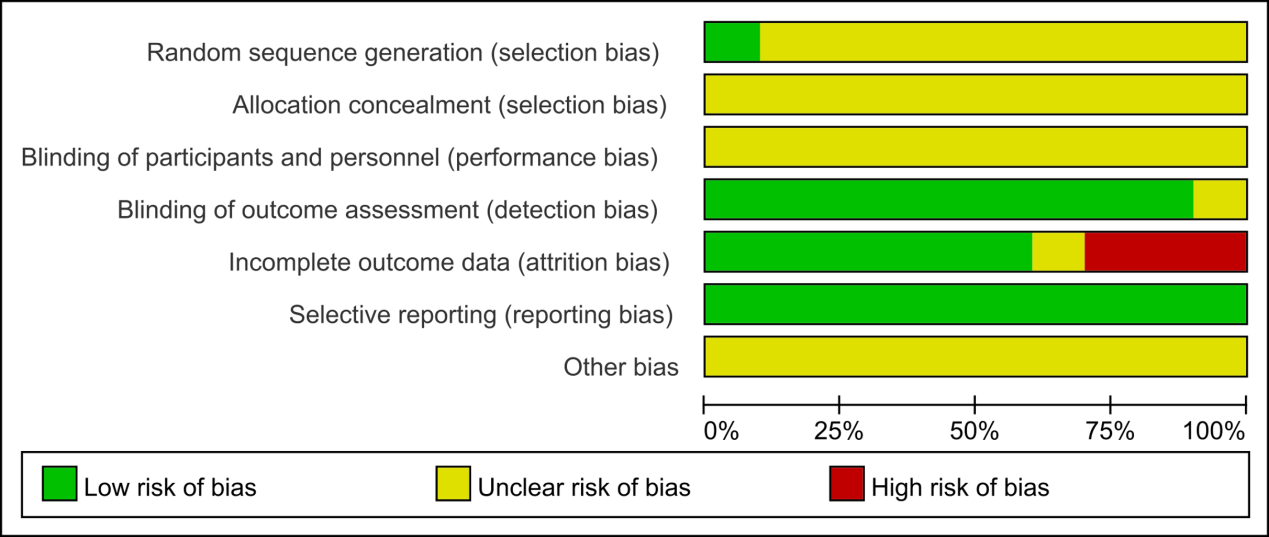


Supplemental material 6: Meta-regression for SBP reduction by control measures

Supplemental material 7: Meta-regression for DBP reduction by control measures

Supplemental material 8: Quality of Evidence


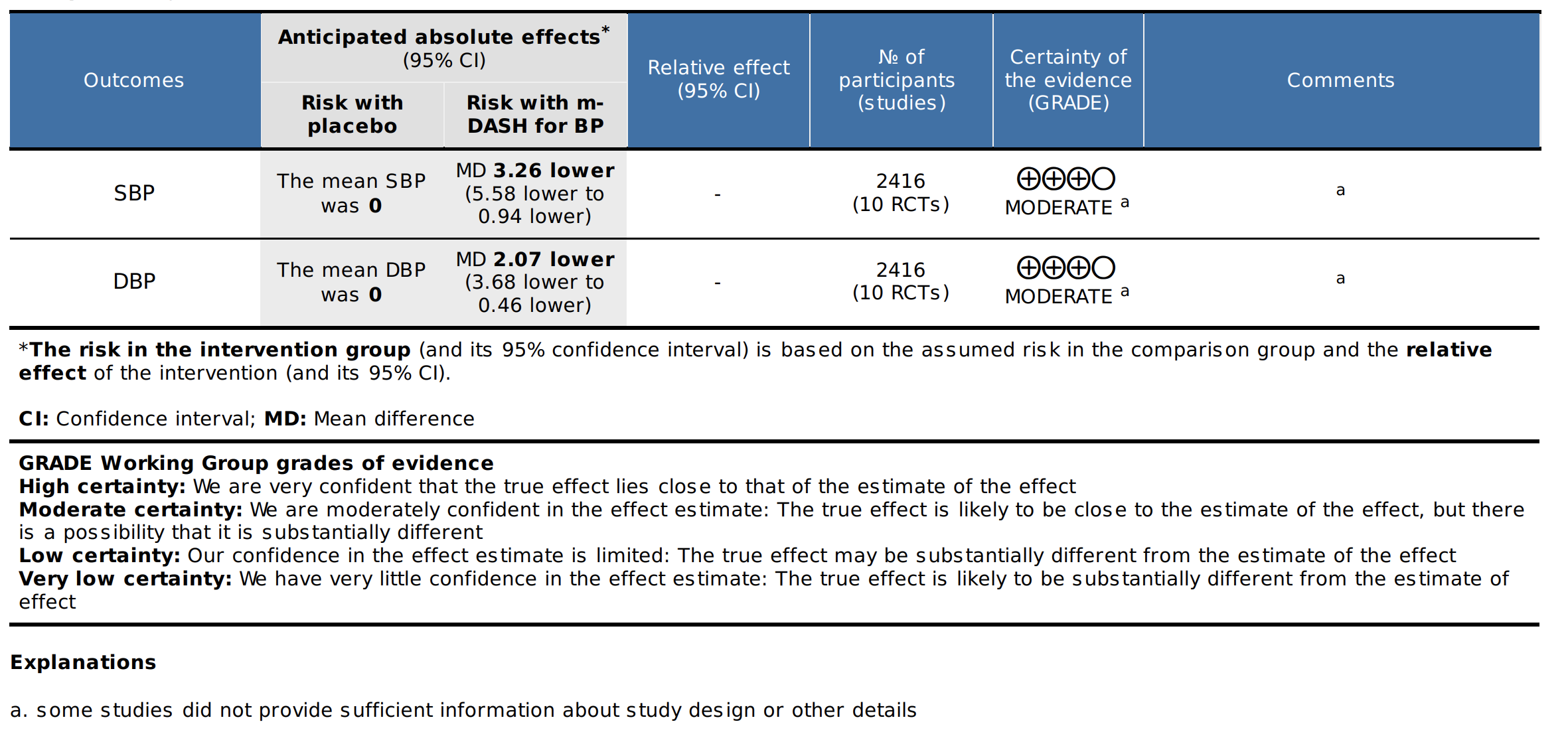

Supplement: Supplementary file 1 [file Table_1.docx]
